# Differences in clinical characteristics and quantitative lung CT features between vaccinated and not vaccinated hospitalized COVID-19 patients in Italy

**DOI:** 10.1186/s13613-023-01103-2

**Published:** 2023-04-03

**Authors:** Davide Chiumello, Alessandro Tavelli, Lorenzo Serio, Sara De Benedittis, Tommaso Pozzi, Roberta Maj, Mara Velati, Serena Brusatori, Rosanna D’Albo, Carmelo Zinnato, Giulia Marchetti, Luigi Camporota, Silvia Coppola, Antonella D’Arminio Monforte

**Affiliations:** 1 Department of Anesthesia and Intensive Care, ASST Santi Paolo e Carlo, San Paolo University Hospital, Via Di Rudini 9, Milan, Italy; 2 Department of Health Sciences, University of Milan, Milan, Italy; 3 Coordinated Research Center on Respiratory Failure, University of Milan, Milan, Italy; 4 Infectious Diseases Unit, Department of Health Sciences, ASST Santi Paolo e Carlo, San Paolo University Hospital, Milan, Italy; 5 Department of Anesthesiology, Medical University of Göttingen, University Medical Center Göttingen, Göttingen, Germany; 6 Department of Anesthesia and Intensive Care, IRCCS San Raffaele Scientific Institute, Milan, Italy; 7 Guy’s and St Thomas’ NHS Foundation Trust, St Thomas’ Hospital, London, SE1 7EH UK

## Abstract

**Background:**

To evaluate the differences in the clinical characteristics and severity of lung impairment, assessed by quantitative lung CT scan, between vaccinated and non-vaccinated hospitalized patients with COVID-19; and to identify the variables with best prognostic prediction according to SARS-CoV-2 vaccination status. We recorded clinical, laboratory and quantitative lung CT scan data in 684 consecutive patients [580 (84.8%) vaccinated, and 104 (15.2%) non-vaccinated], admitted between January and December 2021.

**Results:**

Vaccinated patients were significantly older 78 [69–84] vs 67 [53–79] years and with more comorbidities. Vaccinated and non-vaccinated patients had similar PaO_2_/FiO_2_ (300 [252–342] vs 307 [247–357] mmHg; respiratory rate 22 [8–26] vs 19 [18–26] bpm); total lung weight (918 [780–1069] vs 954 [802–1149] g), lung gas volume (2579 [1801–3628] vs 2370 [1675–3289] mL) and non-aerated tissue fraction (10 [7.3–16.0] vs 8.5 [6.0–14.1] %). The overall crude hospital mortality was similar between the vaccinated and non-vaccinated group (23.1% vs 21.2%). However, Cox regression analysis, adjusted for age, ethnicity, age unadjusted Charlson Comorbidity Index and calendar month of admission, showed a 40% reduction in hospital mortality in the vaccinated patients (HR_adj_ = 0.60, 95%CI 0.38–0.95).

**Conclusions:**

Hospitalized vaccinated patients with COVID-19, although older and with more comorbidities, presented a similar impairment in gas exchange and lung CT scan compared to non-vaccinated patients, but were at a lower risk of mortality.

**Supplementary Information:**

The online version contains supplementary material available at 10.1186/s13613-023-01103-2.

## Introduction

Severe acute respiratory syndrome coronavirus 2 (SARS-CoV-2) affected more than 500 million people and caused more than 6 million deaths worldwide [1]. During the pandemic, control measures, such as isolation, social distancing and the use of face masks, were utilized to limit viral transmission, but were ultimately insufficient to alter the course of the pandemic and the mortality rate [2]. Vaccination aims to increase population immunity, prevent severe disease, and reduce the hospitalization risk [3]. Different types of vaccines have been developed, each with up to 90–95% efficacy for preventing COVID-19 and its related mortality [4–6]. Based on these data, in December 2020 a program of vaccination for adults was launched in Europe and in the United States [7, 8].

In Lombardy (Northern Italy) at the end of 2021, 85% of the population aged 20 years or older, had completed the full primary cycle of COVID-19 vaccination (i.e., the ratio between vaccinated to non-vaccinated = 5.6). By the end of 2022, 87.3% of the Lombardy population aged 20 years or older had completed the primary vaccination course, but the remainder of the target population were still unvaccinated almost 2 years after starting the vaccination campaign in Italy [9].

Adequate protection following vaccination requires a competent immune response and breakthrough infection in vaccinated patients has been reported [10–13]. In vaccinated patients the main risk factors associated with hospital admission were the presence of immunosuppression, older age and cardiac or respiratory failure [11, 14–16].

A smaller proportion of vaccinated patients was admitted to hospital or to intensive care (ICU), received mechanical ventilation or experienced disease progression [15–17]. However, the hospital [15–17] and ICU mortality [18–21] were similar between vaccinated and not vaccinated patients and ranged between 6% and 22%.

Morphological analysis of lung CT scan is considered the modality of choice to evaluate the severity, extent of parenchymal disease and the presence of pulmonary vascular alterations. Furthermore, CT scan can easily detect gastrointestinal, renal and brain injury and predict the risk of clinical deterioration [22–26]. In addition, through quantitative lung CT analysis it is possible to accurately compute lung weight, gas volume, and the amount of aerated and non-aerated lung tissue, which have been previously associated with outcome in patients with non-COVID-19 ARDS [27, 28]. Few studies applied quantitative lung CT analysis in mechanically ventilated COVID-19 patients [29, 30] and it is unclear whether vaccination affects the clinical and radiological severity of hospitalized COVID-19 patients and their survival.

This study aims to evaluate the clinical characteristics and the quantitative lung CT features in vaccinated and not vaccinated hospitalized COVID-19 patients on admission. Moreover, this study aims to establish whether clinical and radiological variables can predict outcome according to vaccination status.

## Materials and methods

### Study design

This was a prospective observational cohort study including patients aged 18 years or older, with a positive polymerase chain reaction (PCR) test for SARS-CoV-2 admitted to San Paolo Hospital of Milan, Italy (ASST Santi Paolo Carlo) between January, 1st and December, 31st 2021. Patients who died in the Emergency Department within 24 h from presentation or those who were not hospitalized were excluded. The study was approved by the institutional review board of the ASST Santi Paolo e Carlo Hospital (protocol number 2020/ST/049 and 2020/ST/049bis, 11 March 2020).

### Clinical and laboratory data collection

We collected demographic, laboratory and clinical data at hospital admission (see supplement); lung CT scan within 24 h from admission; physiological and clinical data during ICU admission, as well as the use of monoclonal antibodies, remdesivir, dexamethasone, biological immunomodulatory agents and low molecular weight heparin, and hospital outcome.

### Definition of variables

SARS-CoV-2 vaccination status was categorized as follow: (1) not vaccinated (no dose of SARS-CoV-2 vaccine administered before hospital admission; (2) partial vaccination with only one dose, or two doses but with the second administered less than 14 days prior to admission); (3) fully vaccinated ( patients who received the second dose of vaccination at least 14 days before admission or patients who had received three vaccination doses) [4, 5, 31].

### Lung CT quantitative analysis

Patients underwent lung CT scan acquisition, spontaneously breathing without any non-invasive or invasive ventilatory support. The lung images were processed and analyzed by a dedicated software capable of performing automatic segmentation (Maluna 3.14, Maluna 2020 [32]). The total lung weight, the total lung gas volume, and the amount of the different compartments (not aerated, poorly aerated, normally aerated and over aerated) were computed using a previously described dedicated software (Maluna 3.14, Maluna 2020) [32, 33].

### Statistical analysis

Characteristics of the study participants, assessed at hospital admission, were compared after stratification by SARS-CoV-2 vaccination status and hospital outcome. Qualitative data are reported as frequencies and percentages, whereas continuous data are presented as mean (SD) or median [IQR], as appropriate.

Continuous variables were compared using Mann–Whitney *U* test. Categorical variables were compared by Chi-square test.

The odds ratio (OR) of death was also adjusted for age, to account for differences in age distribution in the two populations that may have different vaccination rates.

Role of SARS-CoV-2 vaccination on quantitative CT-scan-derived lung parameters was analyzed by means of *t* test and linear regression models, unadjusted and adjusted for identified confounders (age, calendar month of admission, chronic lung diseases, ethnicity and sex) (see Additional file 1: Fig. S3A). For the variable including not aerated and poorly aerated fraction of the total lung weight, a quantile regression was adopted.

The probability of ICU admission in fully vaccinated and not vaccinated COVID-19 patients was investigate by a logistic regression model crude and adjusted for age, Charlson Comorbidity Index, PaO_2_/FiO_2_ at admission and month of admission.

Standard survival analysis with Kaplan–Meier curves and log-rank test were used to estimate the probability of in-hospital death. The time-to-event was calculated from the date of hospital admission to the date of death or last day of hospitalisation. We evaluated the possible association between the different CT-scan lung parameters and time to in-hospital death using unadjusted and adjusted Cox-proportional hazard regression models for each parameter. To investigate this association, we dichotomized the continuous variables of CT-scan using ROC curves and Youden Index (J) method to identify the optimal cutoff for the in-hospital death endpoint. The following variables were considered as confounders (age, age-unadjusted Charlson Comorbidity Index, ethnicity, inflammation, sex and SARS-CoV-2 vaccination status) using the same approach with previously showed DAG (DAG in Additional file 1: Fig. S3C).

The association between vaccination status and the risk of in-hospital death was evaluated by means of Cox regression models, adjusting for factors identified as confounders (age, age-unadjusted Charlson Comorbidity Index, ethnicity, month of admission) (Directed acyclic diagram, DAG in Additional file 1: Fig. S3B).

## Results

### Clinical characteristics and quantitative lung CT scan data at hospital admission

Among 1016 patients admitted to the hospital in the year 2021, 796 had a lung CT scan on admission, and 684 patients were included in the study (see Additional file 1: Fig. S1); 104 (15.2%) were fully vaccinated and 580 (84.8%) were not vaccinated. Among fully vaccinated patients, 20 (19.2%) completed the full primary vaccination course with ChAdOx1, eight (7.7%) with Ad26.COV2.S, eight (7.7%) with mRNA-1273, and 68 (65.4%), with BNT162b2 vaccine. Eleven patients (10.6% of fully vaccinated, 1.6% of study population) had also a third dose of vaccine: eight with BNT162b2 and three with mRNA-1273.

The baseline characteristics of the population are shown in Table 1.

**Table 1:** Clinical characteristics at hospital admission of not vaccinated and fully vaccinated patients.

	Total *n* = 684	Not vaccinated *n* = 580 (84.8%)	Fully vaccinated *n* = 104 (15.2%)	*p*
*Demographic characteristics*				
Age, years	69 [55–80]	67 [53–79]	78 [69–84]	< 0.001
Male sex, *n* (%)	398 (58.2)	334 (57.6)	64 (61.5)	0.452
Caucasian, *n* (%)	600 (87.7)	502 (86.5)	98 (94.2)	0.566
Latin/hispanic, *n* (%)	24 (3.5)	22 (3.8)	2 (1.9)	
Other, *n* (%)	60 (8.7)	56 (9.7)	4 (3.8)	
*Underlying comorbidities*				
Hypertension, *n* (%)	300 (43.9)	245 (42.2)	55 (52.9)	0.044
Stroke, *n* (%)	33 (4.8)	26 (4.5)	7 (6.7)	0.325
AMI, *n* (%)	70 (10.2)	48 (8.3)	22 (21.1)	< 0.001
Diabetes, *n* (%)	133 (19.4)	104 (17.9)	29 (27.9)	0.018
Cardiovascular diseases *n* (%)	135 (19.7)	96 (16.5)	39 (37.5)	< 0.001
COPD, *n* (%)	64 (9.4)	41 (7.1)	23 (22.1)	< 0.001
History of cancer, *n* (%)	58 (8.5)	38 (6.6)	20 (19.2)	< 0.001
CKD, *n* (%)	48 (7)	35 (6)	13 (12.5)	0.017
Chronic liver disease, *n* (%)	15 (2.2)	9 (1.6)	6 (5.8)	0.007
Age-adjusted Charlson score	3 [1–4]	3 [1–4]	4 [3–6]	< 0.001
*Laboratory data*				
Haemoglobin, g/dL	13.5 [12.0–14.7]	13.7 [12.2–14.9]	12.9 [11.3–14.1]	0.005
CRP, mg/L	52.9 (29.2–84.2)	52.2 [27.1–82.1]	60.6 [35.9–102.8]	0.018
LDH, U/L	293 [235–380]	297 [246–397]	263 [215–338]	0.002
Leukocytes count, 10^3^/µL	6.21 [4.65–8.72]	6.1 [4.51–8.73]	6.64 [4.65–8.7]	0.382
Platelets,10^3^/µL	186 [144–247]	186 [146–239]	183 [135–260]	0.910
D-Dimer, ng/µL	320 [211–616]	314 [214–606]	383 [196–690]	0.562
GPT, U/L	29 [19–49]	30 [20–51]	24 [17–43]	0.086
GOT, U/L	44 [19–49]	44 [34–63]	39 [29–60]	0.086
Creatinine, mg/dL	0.9 [0.7–1.2]	0.8 [0.7–1.1]	1 [0.8–1.5]	< 0.001
Procalcitonin, µg/L	0.17 [0.07–0.63]	0.17 [0.07–0.73]	0.22 [0.18–0.26]	0.636
Ferritin, ng/mL	339 [186–692]	357 [182–708]	314 [305–442]	0.960
*Pharmacological treatments*				
Remdesivir, *n* (%)	174 (25.4)	169 (29.1)	5 (4.8)	< 0.001
Heparin profilaxis, *n* (%)	561 (82)	479 (82.6)	82 (78.8)	0.360
Corticosteroid treatment, *n* (%)	579 (84.6)	495 (85.3)	84 (80.8)	0.233
Biological immunomodulator, *n* (%)	49 (7.2)	42 (7.2)	2 (1.9)	0.024
Monoclonal antibodies, *n* (%)	50 (7.3)	22 (3.8)	28 (26.9)	< 0.001

Continuous variables are expressed as median [IQR] and compared with Mann–Whitney *U* test, while categorical data are expressed as *n* (%) and compared with Chi-square test

*COPD*  chronic obstructive pulmonary disease, *AMI* acute myocardial infarction, *CKD*  chronic kidney disease, *CRP*  C-reactive protein, *LDH*  lactate dehydrogenase, *GOT*  glutamic–oxalacetic transaminase, *GPT*  glutamate–pyruvate transaminase. History of cancer was defined as a cancer diagnosis in the last 5 years

Patients were admitted to the hospital after a median of 5 3–6] days from symptoms onset.

Vaccinated patients were significantly older 78 [69–84] vs 67 [53–79] years and with more comorbidities, such as hypertension (52.9% vs 42.2%), ischemic heart disease (21.1% vs 8.3%), COPD (22.1% vs 7.1%) and chronic kidney disease (12.5% vs 6.0%).

**Fig. 1:**
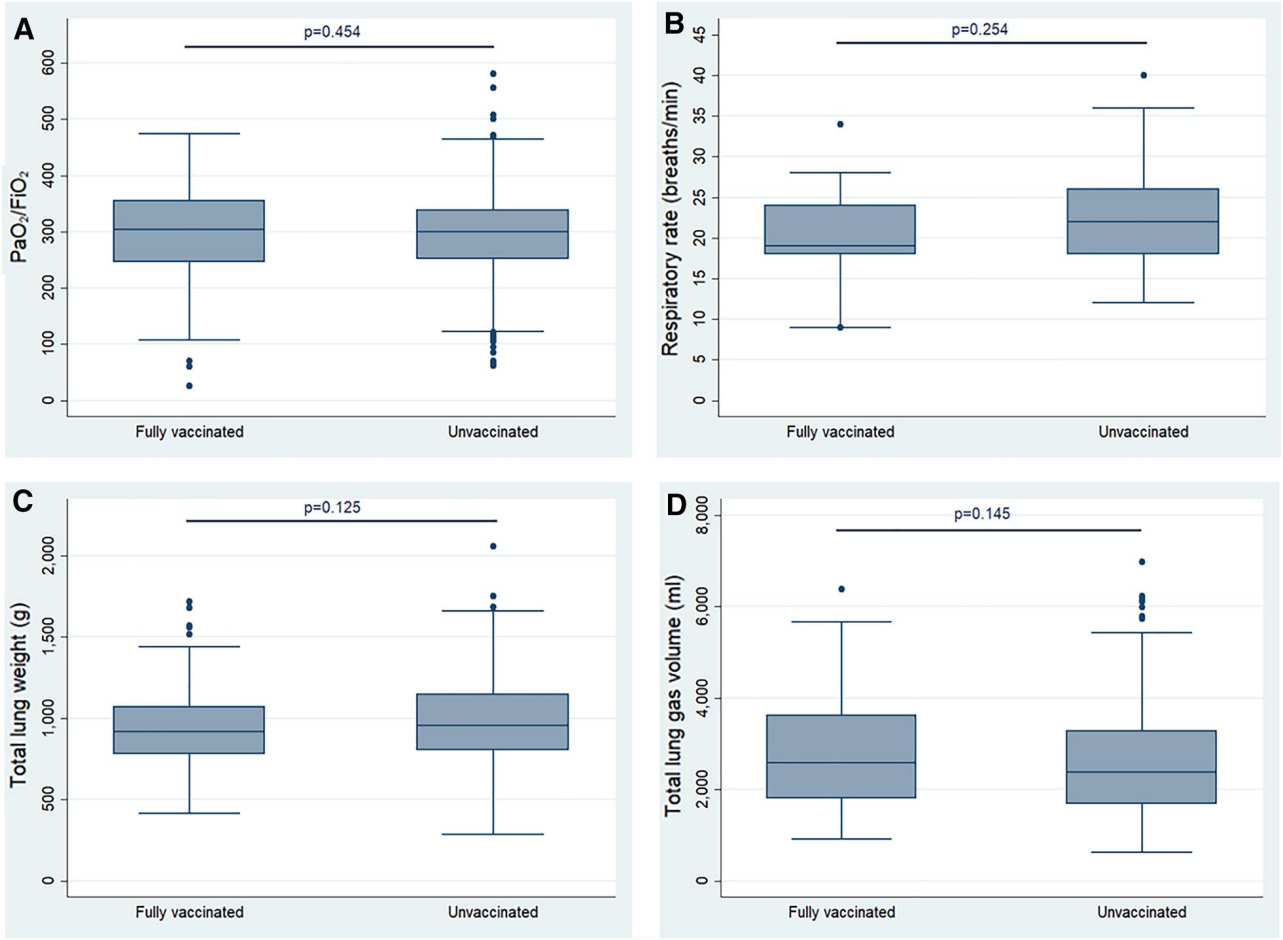
Comparisons between fully vaccinated and not vaccinated (unvaccinated) patients in PaO_2_/FiO_2_ (**A**), respiratory rate (**B**), total lung weight (**C**) and total lung gas volume (**D**). Continuous variables were compared using Mann–Whitney *U* test.

Vaccinated and not vaccinated patients had similar PaO_2_/FiO_2_ (300 [252–342] vs 307 [247–357] mmHg; respiratory rate 22 [8–26] vs 19 [18–26] bpm) (Table 2, Fig. 1); total lung weight (918 [780–1069] vs 954 [802–1149] g), lung gas volume (2579 [1801–3628] vs 2370 [1675–3289] mL) and not aerated tissue fraction (10 [7.3–16.0] vs 8.5 [6.0–14.1] %) (Table 2). In addition, the sum of not aerated and poorly aerated lung tissue fraction was higher in fully vaccinated compared to not vaccinated in the adjusted quantile-regression (36.7% vs 29.3%) (Additional file 1: Table S4A).

**Table 2:** Respiratory function and lung CT scan quantitative data at hospital admission in not vaccinated and fully vaccinated patients

	Total *n* = 684	Not vaccinated *n* = 580 (84.8%)	Fully vaccinated *n* = 104 (15.2%)	*p*
*Respiratory function*				
Respiratory rate, breaths/min	22 [18–26]	22 [8–26]	19 [18–26]	0.254
PaO_2_/FiO_2_ mmHg	300 [252–342]	300 [252–342]	307 [247–357]	0.454
PaO_2_, mmHg	69 [20–81]	68 [60–81]	70 [61–82]	0.631
PaCO_2_, mmHg	33 [30–36]	33 [30–36]	33 [30–37]	0.631
*Lung CT scan quantitative data*				
Total gas volume, ml	2383 [1697–3312]	2370 [1675–3289]	2579 [1801–3628]	0.145
Total lung weight, g	949 [798–1134]	954 [802–1149]	918 [780–1069]	0.125
Over aerated lung weight, %	2 [0.6–5.4]	1.8 [0.5–5.0]	3.5 [1.5–6.9]	< 0.001
Normally aerated lung weight, %	60.3 [51.1–67.5]	60.4 [51.4–67.6]	59.4 [49.3–66.6]	0.432
Poorly aerated lung weight, %	24.8 [18.9–32.5]	25.2 [19.3–32.8]	22.5 [17.9–29.5]	0.043
Not aerated lung weight, %	8.9 [6.2–14.5]	8.5 [6–14.1]	10 [7.3–16.0]	0.031

Continuous variables are expressed as median [IQR] and compared with Mann–Whitney U test.

*FiO*_*2*_  inspired fraction of O_2_, *PaO*_*2*_  arterial partial pressure of O_2_, *PaCO*_*2*_  arterial partial pressure of CO_2_

### Intensive care unit admission

Two fully vaccinated (0.29%) and 26 (4.5%) not vaccinated patients were admitted in the ICU. Fully vaccinated patients had a lower probability of being admitted to ICU compared to not vaccinated after fitting a logistic regression model adjusted for age, Charlson Comorbidity Index, PaO_2_/FiO_2_ at admission and semester of admission (OR = 0.18, 95%CI 0.04–0.85). The comparison in the non-vaccinated group between patients admitted to ICU and those treated outside ICU are reported in Additional file 1: Table S7.

### Outcome

One hundred forty-seven patients (21.5%) died in hospital. Among the non-survivors, 12 (8.1%) were admitted to ICU. The clinical data of the patients according to the hospital outcome is shown in Table 3. Patients who died were significantly older (81 [52–76] vs 64 [74–86] years) and were more likely to have a medical history of hypertension, stroke, myocardial infarction, COPD and cancer. The PaO_2_/FiO_2_ was significantly higher (309 [185–323] vs 255 [266–347] mmHg) in survivors, while the respiratory rate was not different (Table 4). Concerning the lung CT scan data, the lung gas volume was significantly higher, while the lung weight and the percentage of not aerated, and poorly aerated were significantly lower in survivors (Table 4).

**Table 3:** Clinical characteristics at hospital admission of survivors and non survivors patients

	Survivors *n* = 537 (78.5%)	Non survivors *n* = 147 (21.5%)	*p*
*Demographic characteristics*			
Age, years	64 [52–76]	81 [74–86]	< 0.001
Male gender, *n* (%)	314 (58.5)	84 (57.1)	0.772
Caucasian, *n* (%)	459 (85.5)	141 (95.9)	0.012
Latin/hispanic *n* (%)	24 (4.5)	0 (0.0)	
Other, *n* (%)	54 (10.0)	6 (4.1)	
*Underlying comorbidities*			
Hypertension, *n* (%)	217 (40.4)	83 (56.5)	0.001
Stroke, *n* (%)	17 (3.2)	16 (10.9)	< 0.001
AMI, *n* (%)	42 (7.8)	28 (19.0)	< 0.001
Diabetes, *n* (%)	100 (18.6)	33 (22.4)	0.299
Cardiovascular diseases, *n* (%)	88 (16.4)	47 (32.0)	< 0.001
COPD, *n* (%)	39 (7.3)	25 (17.0)	< 0.001
History of cancer, *n* (%)	32 (6.0)	26 (17.7)	< 0.001
CKD, *n* (%)	27 (5.0)	21 (14.3)	< 0.001
Chronic liver disease, *n* (%)	13 (2.4)	2 (1.34	0.437
Age-adjusted Charlson score	2 (1–4)	5 (4–6)	< 0.001
*Laboratory data*			
Haemoglobin, g/dL	13.7 [12.2–14.8]	12.8 [11.1–14.3]	0.009
CRP, mg/L	50.5 [24.9–81.9]	62.1 [38.0–102.7]	0.001
LDH, U/L	287 [232–372]	307 [256–411]	0.035
Leukocytes count, 10^3^/µL	6.16 [4.60–8.41]	6.70 [4.28–10.18]	0.143
Platelets,10^3^/µL	186 [147–242]	191 [134–252]	0.863
D-Dimer, ng/mL	274 [192–553]	476 [289–854]	< 0.001
GPT, U/L	30 [21–53]	23 [17–39]	< 0.001
GOT, U/L	42 [32–61]	47 [36–63]	0.246
Creatinine, mg/dL	0.9 [0.7–1.1]	1.1 (0.8–1.5)	< 0.001
Procalcitonin, µg/L	0.14 [0.05–0.84]	0.20 (0.11–0.57)	0.273
Ferritin, ng/mL	321 [189–634]	469 [117–991]	0.428
*Pharmacological treatments*			
Remdesivir, *n* (%)	148 (27.6)	26 (17.7)	0.015
Heparin profilaxis, *n* (%)	446 (83.0)	116 (78.9)	0.245
Corticosteroid treatment, *n* (%)	447 (83.2)	130 (88.4)	0.124
Biological immunomodulator, *n* (%)	38 (7.1)	12 (8.2)	0.654
Monoclonal antibodies, *n* (%)	50 (7.3)	22 (3.8)	0.180

Continuous variables are expressed as median [IQR] and compared with Mann–Whitney *U* test, while categorical data are expressed as *n* (%) and compared with Chi-square test

*COPD* chronic obstructive pulmonary disease, *AMI*  acute myocardial infarction, *CKD*  chronic kidney disease, *CRP*  C-reactive protein, *LDH*  lactate dehydrogenase, *GOT*  glutamic–oxalacetic transaminase, *GPT*  glutamate–pyruvate transaminase. History of cancer was defined as a cancer diagnosis in the last 5 years

**Table 4:** Respiratory function and lung CT scan quantitative data at hospital admission in survivors and non survivors patients

	Survivors *n* = 537 (78.5%)	Non survivors *n* = 147 (21.5%)	*p*
*Respiratory function*			
Respiratory rate, breaths/min	20 [17–25]	23 [20–56]	0.115
PaO_2_/FiO_2_	309.5 [266.7–347.6]	255.7 [185.7–323.8]	< 0.001
PaO_2_, mmHg	69.0 [61.0–80.4]	68.7 [56.9–84.0]	0.355
PaCO_2_, mmHg	33.5 [30.5–36.7]	32.0 [29.5–36.0]	0.015
*Lung CT scan quantitative data*			
Total gas volume, ml	2476 [1733–3425]	2023 [1435–2623]	< 0.001
Total lung weight, g	938 [792–1121]	991 [810–1202]	0.064
Over aerated lung weight, %	2.2 [0.6–5.8]	1.5 [0.6–4.2]	< 0.020
Normally aerated lung weight, %	61.9 [53.8–68.3]	51.8 [41.4–62.0]	< 0.001
Poorly aerated lung weight, %	23.7 [18.1–30.3]	30.5 [23–39.3]	< 0.001
Not aerated lung weight, %	8.4 [6–12.8]	11.5 [7.1–18.5]	< 0.001

Continuous variables are expressed as median [IQR] and compared with Mann–Whitney *U* test

*FiO*_*2*_  inspired fraction of O_2_, *PaO*_*2*_  arterial partial pressure of O_2_, *PaCO*_*2*_  arterial partial pressure of CO_2_

**Fig. 2:**
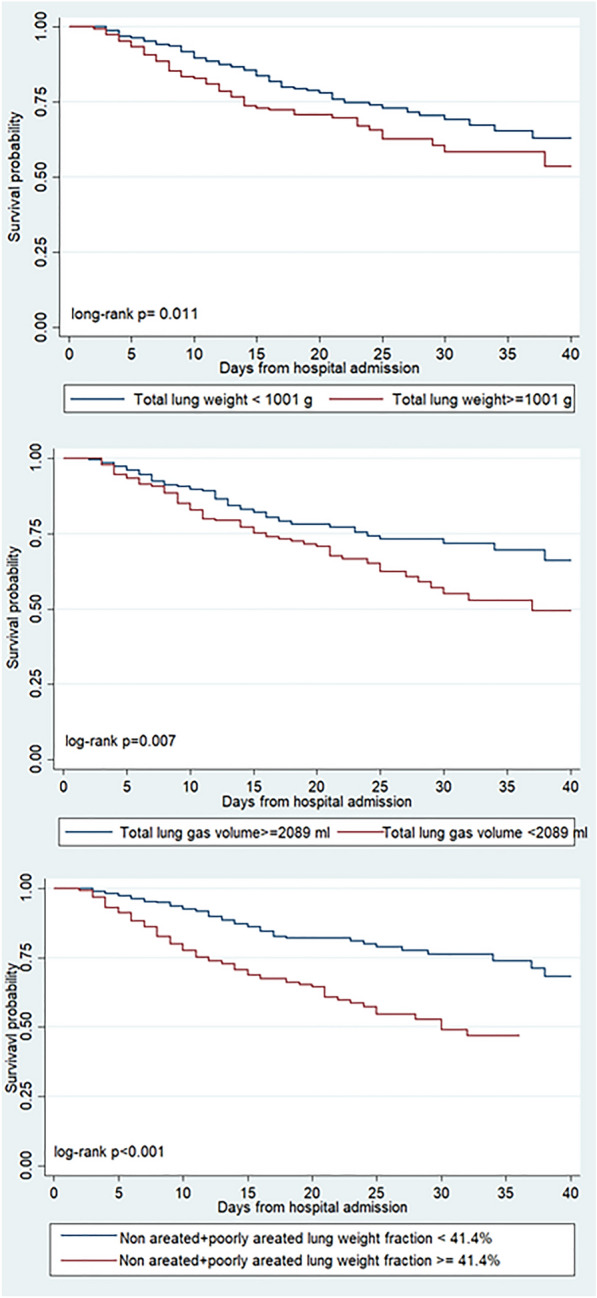
Kaplan–Meier curves according to lung tissue weight (under and above or equal to 1001 g, **A**), to lung gas volume (under and above or equal to 2089 ml, **B**) and to not aerated plus poorly aerated fraction (under and above or equal 41.4%, **C**). The probability of in-hospital death was assessed by log-rank test.

The Kaplan–Meier curves showed a significantly higher cumulative probability of hospital mortality for a lung tissue weight > 1001 g (log-rank *p* = 0.011), for a lung gas volume < 2089 ml (log-rank *p* = 0.007) and for a not aerated plus poorly aerated fraction ≥ 41.4% (log-rank *p* < 0.001) (Fig. 2).

The Cox Regression Models adjusted for age, ethnicity, age-unadjusted Charlson Comorbidity Index, sex, SARS-CoV-2 vaccination status and CRP level, showed a 85% higher risk of hospital mortality for subjects with total lung weight > 1001 g (HR_adj_ = 1.85, 95%CI 1.28–2.68), a 45% higher risk for patients with a lung gas volume < 2089 ml (HR_adj_ = 1.45, 95%CI 1.01–2.08) and a 2.11-fold increase for those with a not aerated plus poorly aerated fraction ≥ 41.4% (95% CI 1.51–2.96) (Additional file 1: Table S5A–C).

The overall crude hospital mortality was not different between fully vaccinated and not vaccinated patients (23% vs 21.2%, *p* = 0.669). The Kaplan Meier curve of hospital survival did not show any significant difference between the two groups (Additional file 1: Fig. S5A, log rank *p* = 0.688). Similarly, the median hospital length of stay was not different (12 [7–20] vs 14 [8–22] days, *p* = 0.57).

However, Cox regression analysis, adjusted for age, ethnicity, age-unadjusted Charlson Comorbidity Index and calendar month of admission, using the not vaccinated group as comparator, fully vaccinated patients showed a statistically significant twofold risk reduction of hospital death (HR_adj_ = 1.96, 95%CI 1.66–2.32) (Additional file 1: Table S5D). Results were confirmed after restricting for the second semester of 2021, where the main circulating variant of concern was Delta (HR_adj_ = 2.25, 95%CI 1.10–4.60) (Additional file 1: Table S5E).

## Discussion

The main findings of this observational study in hospitalized COVID-19 patients were: (1) fully vaccinated patients had a similar degree of lung involvement but were older and with more comorbidities compared to patients who were not vaccinated; (2) the crude hospital and ICU mortality rates were not different among fully vaccinated and not vaccinated patients; and (3) the adjusted hospital mortality rate was significantly lower in fully vaccinated patients.

In Lombardy, up to 89% of the population had been fully vaccinated mainly with mRNA vaccines Comirnaty (BioNTech-Pfizer) and Spikevax (Moderna). It has been reported that the US SARS-CoV-2 vaccination programme was able to prevent up to 56% of all expected hospitalization from December 2020 to September 2021 [3]. Vaccinated patients requiring hospital admission were mainly older patients with comorbidities [11, 12, 14–16].

Although the vaccine efficacy decreases with time, it can remain high (> 70%) at 6 months [12]. In addition, with the emergence of new SARS-CoV-2 variants a reduction in the risk of hospital admission with the variant Omicron compared with the Delta infection was observed [34]. This risk reduction was similar among the fully vaccinated and not vaccinated patients [34]. In this study we observed that there was a significantly lower risk for hospital admission in fully vaccinated patients.

At hospital admission, fully vaccinated patients were older with a higher prevalence of comorbidities compared to not vaccinated patients.

However, the gas exchange (i.e., the PaO_2_/FiO_2_, PaCO_2_) and the respiratory rate were not different between the two populations, suggesting a similar degree of impairment in lung function. In contrast, Tenforde et al., recruiting only patients admitted in hospital with hypoxemia, found a lower number of cases in fully vaccinated compared to not vaccinated (13.5% vs 28.2%) [10]. Regardless of the vaccination status, the current population had on average a similar respiratory rate but a better oxygenation compared to the hospitalized patients during the first wave [35–37].

Concerning the morphological lung abnormalities, the most common non-specific findings in hospitalized patients with COVID-19 included multifocal consolidation and ground glass opacities mainly located in the subpleural space, with a peak of severity within the first week of hospital admission [22, 38]. Applying a morphological analysis, Balacchi et al. did not show any difference regarding the CT pattern abnormality between the first and second wave of the pandemic [39, 40].

To quantify the lung involvement, a CT severity score was proposed which is based on the sum of all the individual lobar score (each lobar score can range from 0 to 5) [41]. According to this score, the lung involvement is categorized as mild, moderate or severe for a score lower than 7, between 8 and 17 and higher than 18, respectively. Fatima et al., reported a similar proportion of patients with moderate and severe lung severity score in fully vaccinated and not vaccinated patients (32% vs 28% and 42% vs 46%, respectively) [41]. Alternatively, the quantitative analysis previously applied in patients with acute respiratory failure, although time consuming and requiring a dedicated software, is considered the best standard lung imaging technique [27, 42]. This analysis is able to accurately assess the lung gas volume, lung weight and the different lung regions (from not aerated to hyperinflated), which are associated with the severity of lung impairment, degree of hypoxemia and hospital outcome [27–29]. To date, there is no study which has evaluated the possible difference in the lung characteristics and impairments in spontaneous breathing fully vaccinated and not vaccinated patients. Our study shows that the lung gas volume, lung weight and total amount of not aerated and poorly aerated lung regions were similar between the two groups. Moreover, in fully vaccinated patients the amount of the sum of not aerated and poorly aerated lung regions were on average less than 34%, suggesting a minimal presence of lung disease. Previous studies in not vaccinated mechanically ventilated COVID-19 ARDS patients reported both a higher lung weight and a greater extension of the non-aerated and poorly aerated lung regions, compared to our study population [30, 43, 44].

### Hospital and intensive care outcome

Since the beginning of the pandemic, the mortality rate has decreased to less than 30% in hospitalized patients [10, 15, 17, 45]. We observed that non-survivors were significantly older, presented with a higher number of comorbidities and had a lower lung gas volume with a higher proportion of non-aerated and poorly aerated lung regions, suggesting a higher impairment and extension of lung abnormalities.

Concerning the hospital mortality rate, we did not find any difference among the two groups (fully vaccinated 21.2% vs not vaccinated 23.1%). Similarly, observational studies analyzing hospitalized patients with COVID-19 during the period between February and November 2021 reported that the mortality rate was not different for vaccinated and not vaccinated [45].

These data may suggest that hospitalized vaccinated patients with COVID-19 showed a similar risk of death compared to not vaccinated. However, vaccinated patients are generally older with a higher prevalence of underlying risk factors than not vaccinated. Thus, in our study, when the risk of death was adjusted for age, the adjusted mortality rate was significantly lower in fully vaccinated patients. Similarly, Tenforde et al. found that the progression to death after COVID-19 hospitalization was associated with a lower likelihood of vaccination and the hospital discharge within 28 days from admission was experienced by a higher number of vaccinated patients when adjusted for age [10].

Considering the whole study population, only a minority was admitted to the intensive care, with less than 5% of not vaccinated patients. Similarly, the ICU outcome was not different between fully vaccinated and not vaccinated patients. The observed mortality rate of the present study was similar to those reported from previous studies [18, 19, 46].

Possible limitations of the present study can be found. This was an observational monocentric study performed in Italy. The determination of the variant of SARS-CoV-2 and the viral load were not performed. Information about other co-infections in the first 48–72 h from hospital admission, which could influence in-hospital mortality, were not collected.

In conclusion, the present data showed that hospitalized vaccinated patients although older and with higher comorbidities, presented a similar impairment both of gas exchange and lung CT scan compared to not vaccinated but a lower risk of mortality.

### Supplementary Information

**Additional file 1: Figure S1**. Study Flowchart. **Figure S2.** Prevalence of vaccination by month of admission 2021. **Table S1**. Outcomes of not vaccinated and fully vaccinated patients. **Table S2.** Maximum Respiratory Support during hospitalization of not vaccinated and fully vaccinated patients. **Table S3.** Maximum Respiratory Support during hospitalization of survivors and not survivors. **Table S4.** Lung CT scan quantitative data after linear regression model adjusted for age, month of admission, presence of chronic lung diseases, ethnicity and sex (A) overall for January–December 2021 and (B) Only for patients admitted in July–December 2021. **Figure S3.** Underlying assumptions regarding the causal links between variables for estimating the: (A) effect of COVID-19 vaccination on lung CT-scan parameters; (B) effect of COVID-19 vaccination on in-hospital death; (C) effect of lung CT-scan parameters on in-hospital death. **Figure S4**. Histograms of fraction of total lung weight. **Table S5.** Unadjusted and adjusted HR from fitting a standard Cox regression model Of in hospital death by (A) total lung tissue mass strata, (B) Lung gas volume strata, (C) Not aerated plus poorly aerated fraction of total lung weight, (D) Vaccination status (January–December 2021), (E) Vaccination status (only July–December 2021). **Table S6.** Probability of admission in ICU in vaccinated and non-vaccinated COVID-19 patients has been investigate by means of logistic regression model crude and adjusted for age, Charlson Comorbidity Index, PaO2/FiO2 at admission and period of admission. **Table S7**. Clinical Characteristic, lung CT scan quantitative data, respiratory function maximum respiratory support of not vaccinated patients admitted or not admitted in ICU. **Figure S5A.** Fully vaccinated and Not vaccinated Kaplan-Meier survival curves. B. Age strata KM survival curves survival.

## Data Availability

The data set used for this study is available from the corresponding author upon reasonable request.

## Abbreviations

SARS-CoV-2: Severe acute respiratory syndrome coronavirus 2
COVID-19: Coronavirus disease 19
ICU: Intensive care unit
CT: Computed tomography
PCR: Polymerase chain reaction
OR: Odds ratio
ROC: Receiver operating characteristics
DAG: Directed acyclic graph
PaO_2_: Arterial oxygen partial pressure
FiO_2_: Inspired oxygen fraction.
