# Supplementary material for: Differences in clinical characteristics and quantitative lung CT features between vaccinated and not vaccinated hospitalized COVID-19 patients in Italy
Source: Ann Intensive Care. 2023 Apr 3;13:24. doi: 10.1186/s13613-023-01103-2 (PMC10068232; doi:10.1186/s13613-023-01103-2)
Supplement: Supplementary file 1 — Additional file 1: Figure S1. Study Flowchart. Figure S2. Prevalence of vaccination by month of admission 2021. Table S1. Outcomes of not vaccinated and fully vaccinated patients. Table S2. Maximum Respiratory Support during hospitalization of not vaccinated and fully vaccinated patients. Table S3. Maximum Respiratory Support during hospitalization of survivors and not survivors. Table S4. Lung CT scan quantitative data after linear regression model adjusted for age, month of admission, presence of chronic lung diseases, ethnicity and sex (A) overall for January–December 2021 and (B) Only for patients admitted in July–December 2021. Figure S3. Underlying assumptions regarding the causal links between variables for estimating the: (A) effect of COVID-19 vaccination on lung CT-scan parameters; (B) effect of COVID-19 vaccination on in-hospital death; (C) effect of lung CT-scan parameters on in-hospital death. Figure S4. Histograms of fraction of total lung weight. Table S5. Unadjusted and adjusted HR from fitting a standard Cox regression model Of in hospital death by (A) total lung tissue mass strata, (B) Lung gas volume strata, (C) Not aerated plus poorly aerated fraction of total lung weight, (D) Vaccination status (January–December 2021), (E) Vaccination status (only July–December 2021). Table S6. Probability of admission in ICU in vaccinated and non-vaccinated COVID-19 patients has been investigate by means of logistic regression model crude and adjusted for age, Charlson Comorbidity Index, PaO2/FiO2 at admission and period of admission. Table S7. Clinical Characteristic, lung CT scan quantitative data, respiratory function maximum respiratory support of not vaccinated patients admitted or not admitted in ICU. Figure S5A. Fully vaccinated and Not vaccinated Kaplan-Meier survival curves. B. Age strata KM survival curves survival. [file 13613_2023_1103_MOESM1_ESM.docx]

**Additional Content**

**Figure S1. Study Flowchart**

**
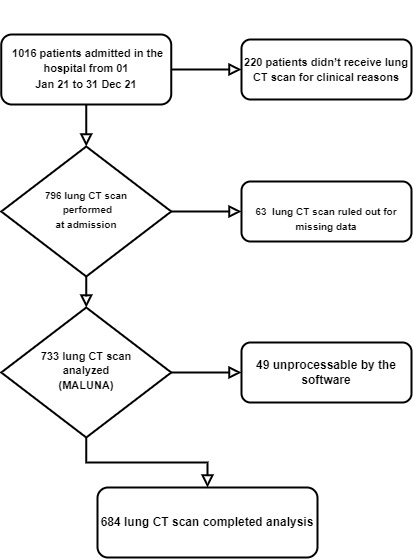
**

**Figure S2. Prevalence of vaccination by month of admission 2021**

**Table S1**. Outcomes of not vaccinated and fully vaccinated patients

|  | Total  n=684 | Not vaccinated  n=580 (84.8%) | Fully vaccinated  n=104 (15.2%) | *P* |
| --- | --- | --- | --- | --- |
| Outcomes |  |  |  |  |
| Time from symptoms onset and hospitalization, days | 5 [3-6] | 5 [3-8] | 5 [2-7] | 0.181 |
| Hospital lenght of stay, days | 13 [7-20] | 12 [7-20] | 14 [8-22] | 0.57 |
| ICU admission, n (%) | 28 (4) | 26 (4.4) | 2 (1.9) |  |
| In hospital mortality, n (%) | 147 (21.5) | 123 (21.2) | 24 (23.1) | 0.669 |

ICU= Intensive Care Unit.

**Table S2**. Maximum Respiratory Support during hospitalization of not vaccinated and fully vaccinated patients

**Table S3**. Maximum Respiratory Support during hospitalization of survivors and not survivors.

|  | Total  n=684 | Not vaccinated  n=580 (84.8%) | Fully vaccinated  n=104 (15.2%) | (P*p* |
| --- | --- | --- | --- | --- |
| Respiratory support, n (%) |  |  |  |  |
| None n | 49 (7.2) | 42 (7.2) | 7 (6.7) | 0.011 |
| O_2_ low flows | 225 (32.9) | 193 (33.3) | 32 (30.8) |  |
| O_2_ high flows | 61 (8.9) | 42 (7.2) | 19 (18.3) |  |
| Helmet CPAP | 247 (36.1) | 217 (37.4) | 30 (28.8) |  |
| NIV | 73 (10.7) | 60 (10.3) | 13 (12.5) |  |
| IMV | 28 (4) | 26 (4.1) | 2 (1.9) |  |

|  | Survivors  N=537 (78.5%) | Not survivors  N=147 (21.5%) | *pp* |
| --- | --- | --- | --- |
| Respiratory support, n (%) |  |  | <0.001 |
| None | 49 (9.1) | 0 (0.0) |  |
| O_2_ low flows | 190 (35.4) | 35 (24.0) |  |
| O_2_ high flows | 57 (10.6) | 4 (2.7) |  |
| Helmet CPAP | 186 (34.6) | 58 (39.7) |  |
| NIV | 38 (7.1) | 37 (25.3) |  |
| IMV | 16 (2.9) | 12 (8.2) |  |

O_2_ low flows= O_2_ requirement < 5L/min; O2 high flows = O_2_ requirement >5 L/min; CPAP= Continous Positive Airway Pressure (Helmet); NIV=Non invasive ventilation; IMV= Invasive mechanical ventilation

*Role of COVID-19 vaccination on quantitative CT-scan derived lung parameters (total lung volume, lung tissue mass, lung gas volume) have been analysed by means of t-test and linear regression models unadjusted and adjusted for the identified confounders (age, calendar month of admission, chronic lung diseases, ethnicity and sex). Over plus normal aerated fraction of total lung weight and Not plus poorly aerated fraction of total lung weight – are not normally distribuited and have been evaluated with Wilcoxon rank-sum test in the crude analysis and with quantile regression for the adjusted model for the same set of confounders*

**Table S4**. Lung CT scan quantitative data after linear and quantile regression models adjusted for age, month of admission, presence of chronic lung diseases, ethnicity and sex (A) overall for January-December 2021 and (B) Only for patients admitted in July-December 2021.

**(A) Juanary-December 2021**

|  | Unadjusted | | | | Adjusted* | | |
| --- | --- | --- | --- | --- | --- | --- | --- |
|  | **Unvaccinated**  **n=580** | **Fully vaccinated**  **n=104** | **P**  **(t-test)** | | **Unvaccinated**  **n=580** | **Fully vaccinated**  **n=104** | **p (linear regression)** |
| Total lung volume, mean SD, (mL) | 3544.7 ±1236.97 | 3658.3  ±1231.5 | 0.388 | | 3517  (3426-3609) | 3808  (3539-4077) | 0.060 |
| Total lung weight, mean SD (g) | 982.3  ± 248.7 | 943.2  ± 259.4 | 0.143 | | 980  (962-999) | 953.8  (899-1007) | 0.392 |
| Total gas volume, mean SD (mL) | 2564  ±1130.5 | 2717  ±1113.2 | 0.203 | 2539  (2452-2626) | | 2856  (2599-3112) | 0.032 |
|  | **Unvaccinated**  **n=580** | **Fully vaccinated**  **n=104** | **P**  **(t-test)** | **Unvaccinated**  **n=580** | | **Fully vaccinated**  **n=104** | **p (quantile regression)** |
| Over plus normal aerated fraction of total lung weight, %, median (95%CI) | 64.0  (53.3-72.9) | 66.1  (51.7-72.9) | 0.078 | | 63.3  (61.9-64.8) | 70.7  (66.5-74.9) | 0.002 |
| Not plus poorly aerated fraction of total lung weight %, median (95%CI) | 36.0  (27.1-46.6) | 66.1  (27.1-48.3) | <0.001 | | 36.7  (35.2-38.1) | 29.3  (25.1-33.5) | 0.002 |

**(B) July-December 2021**

|  | Unadjusted | | | | Adjusted* | | |
| --- | --- | --- | --- | --- | --- | --- | --- |
|  | **Unvaccinated**  **n=580103** | **Fully vaccinated**  **n=104102** | **P**  **(t-test)** | | **Unvaccinated**  **n=580103** | **Fully vaccinated**  **n=104102** | **p (linear or quantile regression)** |
| Total lung volume, mean SD, (mL) | 3322.4  ± 3106.1 | 3680.1  ± 3438.3 | 0.030 | | 3329.3  3104.6-3554.0 | 3673.2  3447.2-3899.1 | 0.048 |
| Total lung weight, mean SD (g) | 956.9  ± 290.5 | 945.3  ± 261.2 | 0.763 | | 955.5  907.9-1003.2 | 946.7  898.7-994.6 | 0.809 |
| Total gas volume, mean SD (mL) | 2367.5  ± 993.1 | 2736.8  ± 1111.1 | 0.013 | 2375.8  2164.4-2587.1 | | 2728.5  2515.9-2941.0 | 0.031 |
|  | **Unvaccinated**  **n=580** | **Fully vaccinated**  **n=104** | **P**  **(t-test)** | **Unvaccinated**  **n=580** | | **Fully vaccinated**  **n=104** | **p (quantile regression)** |
| Over plus normal aerated fraction of total lung weight, %, median (95%CI) | 59.8  (56.0-65.7) | 66.1  (62.1-70.8) | 0.115 | | 60.2 |  | 60.2 |
| Not plus poorly aerated fraction of total lung weight %, median (95%CI) | 40.1  (34.3-44.0) | 33.8  (29.2-37.8) | 0.115 | | (56.6-63.8) | 66.7 | (56.6-63.8) |

**Adjusted for age, calendar_month, chronic_lung_dis, comorbidities, ethnicity/ita*

**Figure S3.** Underlying assumptions regarding the causal links between variables for estimating the: (A) effect of COVID-19 vaccination on lung CT-scan parameters; (B) effect of COVID-19 vaccination on in-hospital death; (C) effect of lung CT-scan parameters on in-hospital death.

**(A)**


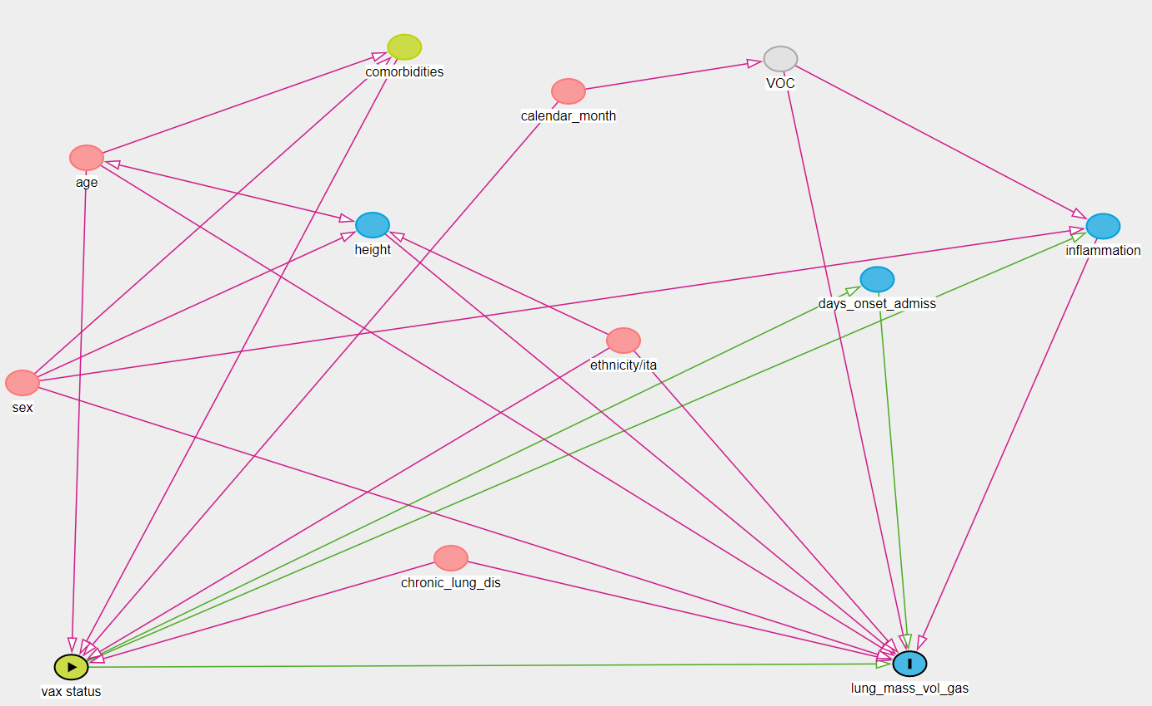


**(B)**


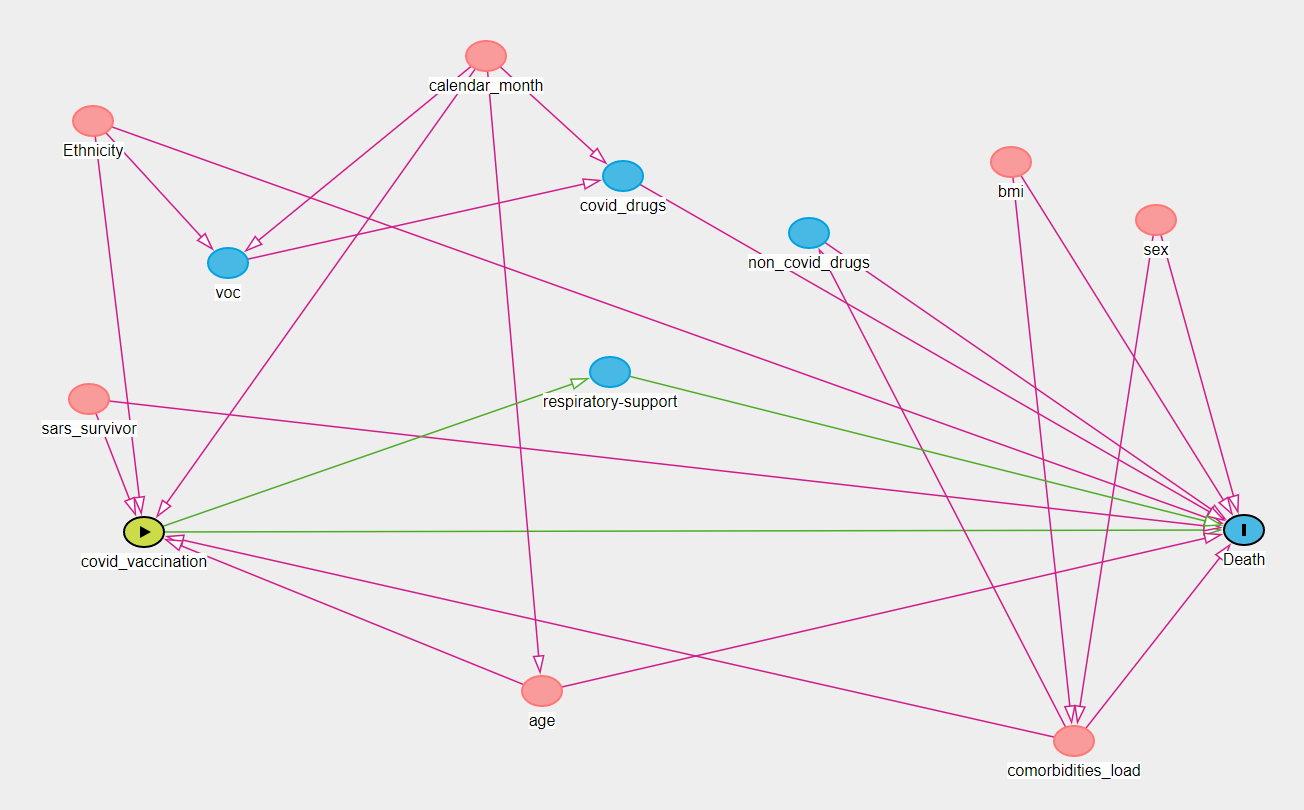


**(C)**


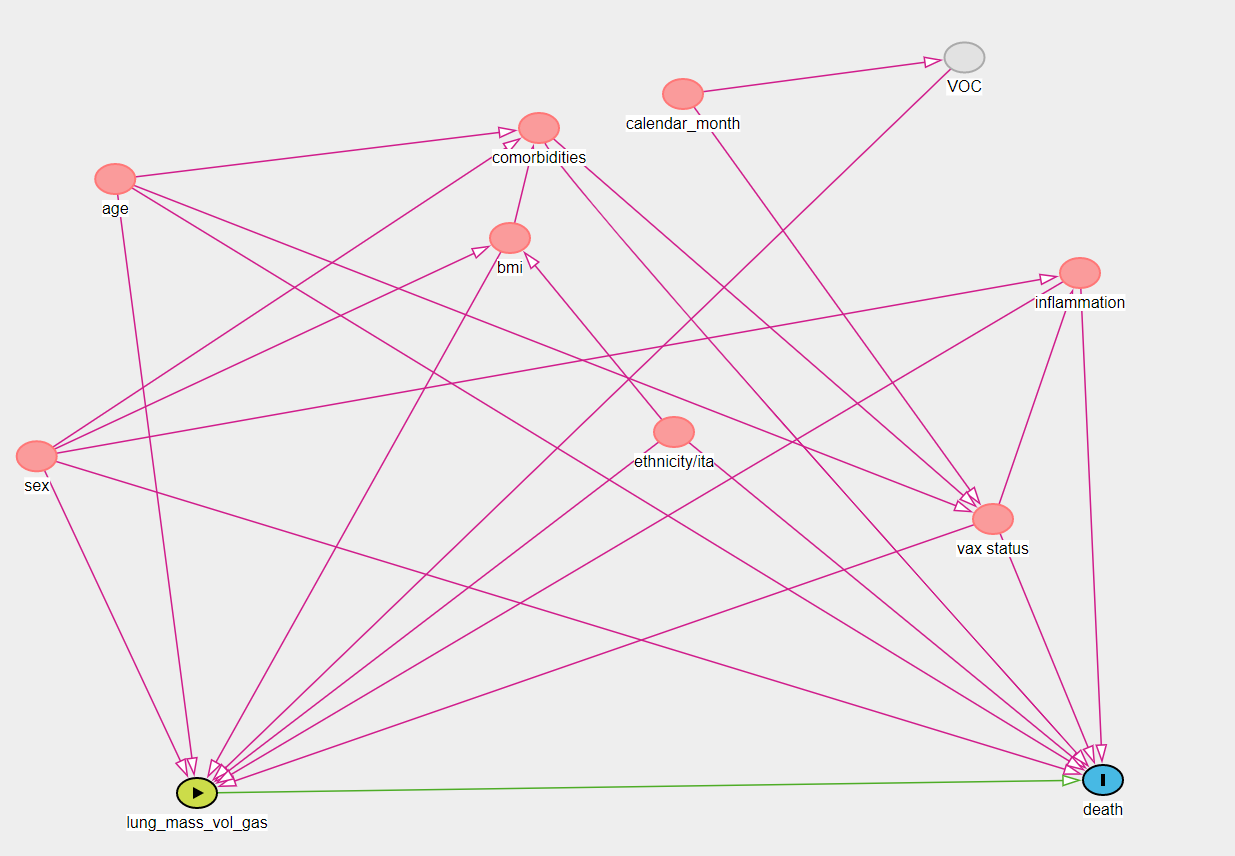


**Figure S4.** Histograms of fraction of total lung weight.

a) overaerated, normally aerated, poorly-aerated, not aerated fraction of total lung weight.

b) %tot. weight aerated= Over plus normal aerated fraction of total lung weight; % tot. weight non-aerated= Not plus poorly aerated fraction of total lung weight.

**Table S5.** Unadjusted and adjusted HR from fitting a standard Cox regression model Of in hospital death by (A) total lung tissue mass strata, (B) Lung gas volume strata, (C) Not aerated plus poorly aerated fraction of total lung weight, (D) Vaccination status (Janurary-Decembert 2021), (E) Vaccination status (only July-December 2021).

|  | **Unadjusted** | | | | **Adjusted Model** | | | |
| --- | --- | --- | --- | --- | --- | --- | --- | --- |
| **(A) Total Lung Tissue Mass** | **HR** | **p** | **95%CI** |  | **AHR** | **p** | **95%CI** |  |
| **Total Tissue Mass > 1001g** | **1.50** | **0.010** | **1.09** | **2.08** | **1.85** | **0.001** | **1.28** | **2.68** |
| Age, per 10 years older | 1.91 | <0.001 | 1.64 | 2.23 | 2.04 | <0.001 | 1.71 | 2.43 |
| Caucasian (vs other Ethnicity) | 3.12 | 0.006 | 1.38 | 7.06 | 0.74 | 0.494 | 0.31 | 1.77 |
| Age-unadj CCI, per 1 pt more | 1.15 | 0.053 | 1.00 | 1.31 | 1.08 | 0.048 | 1.00 | 1.16 |
| Gender, Male (vs Female) | 0.98 | 0.926 | 0.71 | 1.37 | 0.97 | 0.893 | 0.67 | 1.42 |
| COVID vaccine 2+ doses (vs no/1d) | 1.03 | 0.893 | 0.67 | 1.60 | 0.67 | 0.088 | 0.42 | 1.06 |
| CRP > 60 mg/L | 1.57 | 0.018 | 1.08 | 2.28 | 1.51 | 0.033 | 1.03 | 2.19 |

| **(B) Lung Gas Volume** | **HR** | **p** | **95%CI** |  | **AHR** | **p** | **95%CI** |  |
| --- | --- | --- | --- | --- | --- | --- | --- | --- |
| **Lung Gas Volume < 2089 ml** | **1.54** | **0.008** | **1.12** | **2.14** | **1.45** | **0.043** | **1.01** | **2.08** |
| Age, per 10 years older | 1.91 | <0.001 | 1.64 | 2.23 | 1.92 | <0.001 | 1.62 | 2.29 |
| Caucasian (vs other Ethnicity) | 3.12 | 0.006 | 1.38 | 7.06 | 0.93 | 0.866 | 0.38 | 2.25 |
| Age-unadj CCI, per 1 pt more | 1.15 | 0.053 | 1.00 | 1.31 | 1.08 | 0.046 | 1.00 | 1.16 |
| Gender, Male (vs Female) | 0.98 | 0.926 | 0.71 | 1.37 | 1.44 | 0.050 | 1.00 | 2.07 |
| COVID vaccine 2+ doses (vs no/1d) | 1.03 | 0.893 | 0.67 | 1.60 | 0.60 | 0.030 | 0.38 | 0.95 |
| CRP > 60 mg/L | 1.57 | 0.018 | 1.08 | 2.28 | 1.66 | 0.008 | 1.14 | 2.42 |

| **(C) Not aerated + poorly aerated fraction** | **HR** | **p** | **95%CI** |  | **AHR** | **p** | **95%CI** |  |
| --- | --- | --- | --- | --- | --- | --- | --- | --- |
| **Not aerated + poorly aerated fraction ≥ 41.4%** | **2.48** | **<0.001** | **1.78** | **3.46** | **2.11** | **<0.001** | **1.51** | **2.96** |
| Age, per 10 years older | 1.91 | <0.001 | 1.64 | 2.23 | 1.89 | <0.001 | 1.59 | 2.24 |
| Caucasian (vs other Ethnicity) | 3.12 | 0.006 | 1.38 | 7.06 | 0.93 | 0.864 | 0.38 | 2.24 |
| Age-unadj CCI, per 1 pt more | 1.15 | 0.053 | 1.00 | 1.31 | 1.08 | 0.037 | 1.00 | 1.17 |
| Gender, Male (vs Female) | 0.98 | 0.926 | 0.71 | 1.37 | 1.25 | 0.198 | 0.89 | 1.76 |
| COVID vaccine 2+ doses (vs no/1d) | 1.03 | 0.893 | 0.67 | 1.60 | 0.60 | 0.029 | 0.38 | 0.95 |
| CRP > 60 mg/L | 1.57 | 0.018 | 1.08 | 2.28 | 1.43 | 0.064 | 0.98 | 2.09 |

| **(D) Vaccination Status (Jan-Dec 2021)** | **HR** | **p** | **95%CI** |  | **AHR** | **p** | **95%CI** |  |
| --- | --- | --- | --- | --- | --- | --- | --- | --- |
| COVID vaccine 2+ doses (vs no/1dose) | 1.03 | **0.893** | 0.67 | 1.60 | 2.07 | **0.021** | 1.12 | 3.86 |
| Caucasian (vs other Ethnicity) | 3.12 | **0.006** | 1.38 | 7.06 | 0.84 | 0.699 | 0.35 | 2.03 |
| Age-unadj CCI, per 1 pt more | 1.14 | **<0.001** | 1.08 | 1.20 | 1.10 | **0.010** | 1.02 | 1.18 |
| Month of admission, per 1 more | 0.99 | 0.668 | 0.95 | 1.04 | 1.04 | 0.172 | 0.98 | 1.11 |
| Age, per 10 years older | 1.91 | **<0.001** | 1.64 | 2.23 | 1.96 | **<0.001** | 1.66 | 2.32 |

| **(E) Vaccination Status (Jul-Dec 2021)** | **HR** | **p** | **95%CI** |  | **AHR** | **p** | **95%CI** |  |
| --- | --- | --- | --- | --- | --- | --- | --- | --- |
| COVID vaccine 2+ doses (vs no/1dose) | 1.37 | 0.326 | 0.73 | 2.59 | 2.26 | **0.025** | 1.11 | 4.60 |
| Caucasian (vs other Ethnicity) |  |  |  |  | . | . | . | . |
| Age-unadj CCI, per 1 pt more | 1.15 | 0.053 | 1.00 | 1.31 | 1.02 | 0.851 | 0.84 | 1.23 |
| Month of admission, per 1 more | 1.39 | **0.006** | 1.10 | 1.75 | 1.42 | **0.004** | 1.12 | 1.80 |
| Age, per 10 years older | 1.94 | **<0.001** | 1.41 | 2.67 | 2.08 | **<0.001** | 1.47 | 2.94 |

**Table S6.** The probability of admission in ICU in vaccinated and non-vaccinated COVID-19 patients has been investigate by means of logistic regression model crude and adjusted for age, Charlson Comorbidity Index, PaO2/FiO2 at admission and period of admission.

|  | **OR (95%CI)** | ***p*** | **AOR (95%CI)** | ***p*** |
| --- | --- | --- | --- | --- |
| COVID-19 Fully vaccinated/unvaccinated | 0.42 (0.10-1.79) | 0.24 | 0.18 (0.04-0.85) | 0.031 |
| Age, years, per 10 older | 0.74 (0.60-0.92) | 0.01 | 0.81 (0.61-1.07) | 0.133 |
| Age Unadjusted Charlson Index, per 1 pt more | 0.48 (0.25-0.91) | 0.03 | 0.61 (0.31-1.21) | 0.161 |
| PaO2/FiO2 admission, for 10 more | 0.95 (0.91-0.99) | 0.01 | 0.94 (0.89-0.98) | 0.005 |
| 2nd semester 2021 (vs 1st) | 4.51 (2.05-9.96) | <0.001 | 8.89 (3.72-2.25) | <0.001 |

**Table S7**. Clinical Characteristic, lung CT scan quantitative data, respiratory function maximum respiratory support of not vaccinated patients admitted or not admitted in ICU.

|  | Total not vaccinated  n=580 | Not ICU admitted  n=554 | Icu admitted  n=26 | p |
| --- | --- | --- | --- | --- |
| Demographic characteristics |  |  |  |  |
| Age, years | 67 [53-79] | 67 [53-79] | 62 [52-68] | 0.0262 |
| Male gender n % | 334 (57) | 318 (57) | 16 (61) | 0.67 |
| Caucasian n % | 502 (86) | 479 (86) | 23 (89) | 0.97 |
| Latin n % | 22 (48) | 21 (4) | 1 (4) |  |
| Other n % | 56 (10) | 54 (10) | 2 (7) |  |
| Charlson Score age adjusted | 3 [1-4] | 3 [1-4] | 2 [1-2] | 0.0669 |
| Underlying Comorbidities, n. % |  |  |  |  |
| Cardiovascular disease | 96 (16) | 93 (16) | 3 (11) | 0.48 |
| Cerebrovasculsar disease | 26 (4) | 26 (5) | 0 | 0.258 |
| COPD | 41 (7) | 40 (7) | 1 (3) | 0.512 |
| Hypertension | 245 (42) | 233 (42) | 12 (46) | 0.67 |
| Diabetes | 104 (18) | 102 (18) | 2 (7) | 0.164 |
| IMA | 48 (8) | 45 (8) | 3 (11) | 0.537 |
| Stroke | 26 (4) | 26 (5) | 0 | 0.258 |
| Cancer | 38 (6) | 38 (6) | 0 | 0.167 |
| CKD | 35 (6) | 35 (6) | 0 | 0.168 |
| Chronic liver disease | 9 (2) | 9 (2) | 0 | 0.512 |
| Respiratory function |  |  |  |  |
| PaO_2_/FiO_2_ | 300 [252-343] | 301 [253-342] | 271 [135-300] | 0.0032 |
| PaO_2_ mmHg | 68 [60-81] | 69 [60-80] | 63 [57-72] | 0.089 |
| PaCO2 mmHg | 33 [30-36] | 33 [30-36] | 31 [28-34] | 0.016 |
| RR bpm | 22 [18-26] | 22 [18-25] | 30 [16-30] | 0.0225 |
| Respiratory support |  |  |  |  |
| None n. % | 43 (7) | 43 (7) | 0 | 0.001 |
| Low flow O_2,_ n. % | 193 (34) | 193 (33) | 0 |  |
| High flow O_2_ n. % | 42 (7) | 42 (7) | 0 |  |
| PEEP n. % | 214 (38) | 214 (36) | 0 |  |
| NIV n. % | 36 (6) | 36 (7) | 0 |  |
| IMV n. % | 26 (5) | 0 | 26 (100) | 0.001 |
| Main Laboratory data |  |  |  |  |
| PCR mg/L | 52 [26-82] | 51 [25-82] | 56 [39-96] | 0.114 |
| LDH U/L | 297 [246-395] | 292 [242-383] | 436 [354-554] | 0.000 |
| Lymphocities 10^3/µL | 1.01 [0.68-1.41] | 1.02 [0.68-1.41] | 0.84 [0.67-1.17] | 0.1116 |
| Lung CT scan quantitative data |  |  |  |  |
| Total gas volume mL | 2370 [1675-3289] | 2388 [1697-3298] | 1845 [1432-2572] | 0.0358 |
| Total lung weight g | 954 [801-1149] | 949 [799-1135] | 1103 [880-1259] | 0.0281 |
| Treatment administered  n. % |  |  |  |  |
| Heparin | 479 (82) | 453 (81) | 26 (100) | 0.017 |
| Steroid | 494 (85) | 470 (84) | 24 (92) | 0.295 |

*PCR= C-reactive protein; LDH= lactate dehydrogenase; COPD= Chronic Obstructive Pulmonary Disease, IMA= ischemic myocardial infarction, CKD= Chronic Kidney disease . History of cancer was defined as a cancer diagnosis in the last 5 years.* FiO_2_ = Inspired fraction of O_2_; PaO_2_= Arterial partial pressure of O_2;_ PaCO_2_ = Arterial partial pressure of CO_2_.

*Continuous variables are expressed as median [IQR] and compared with Mann-Whitney U test, while categorical data are expressed as n (%) and compared with Chi-square test.*

Standard survival analysis as Kaplan–Meier curves and log-rank test have been used to estimate the probability of in-hospital death. The time-to event has been calculated from the date of hospital admission to the date of death or last day of hospitalisation.

**Supplementary Figure S5A**. Fully vaccinated and Not vaccinated Kaplan Meier survival curves.

Log-rank p= 0.06887

A

Not vaccinated=Vax_2more_doses=0; Fully vaccinated= vax_2more_doses=1

**Figure S5B.** Age strata Kaplan Meier survival curves

Log-rank p=<0.001

B
